# Mechanisms of Perivascular Adipose Tissue Dysfunction in Obesity

**DOI:** 10.1155/2013/402053

**Published:** 2013-11-07

**Authors:** Maria S. Fernández-Alfonso, Marta Gil-Ortega, Concha F. García-Prieto, Isabel Aranguez, Mariano Ruiz-Gayo, Beatriz Somoza

**Affiliations:** ^1^Instituto Pluridisciplinar and Facultad de Farmacia, Universidad Complutense, Juan XXIII 1, 28040 Madrid, Spain; ^2^Departamento de Ciencias Farmacéuticas y de la Salud, Facultad de Farmacia, Universidad CEU-San Pablo, 28660 Madrid, Spain

## Abstract

Most blood vessels are surrounded by adipose tissue. Similarly to the adventitia, perivascular adipose tissue (PVAT) was considered only as a passive structural support for the vasculature, and it was routinely removed for isolated blood vessel studies. In 1991, Soltis and Cassis demonstrated for the first time that PVAT reduced contractions to noradrenaline in rat aorta. Since then, an important number of adipocyte-derived factors with physiological and pathophysiological paracrine vasoactive effects have been identified. PVAT undergoes structural and functional changes in obesity. During early diet-induced obesity, an adaptative overproduction of vasodilator factors occurs in PVAT, probably aimed at protecting vascular function. However, in established obesity, PVAT loses its anticontractile properties by an increase of contractile, oxidative, and inflammatory factors, leading to endothelial dysfunction and vascular disease. The aim of this review is to focus on PVAT dysfunction mechanisms in obesity.

## 1. Introduction

Obesity is an independent risk factor for the development of endothelial dysfunction and vascular disease, hypertension, myocardial infarction, and stroke [1]. The probability of suffering from vascular diseases is four times higher in obese (body mass index >30 kg/m^2^) than in normal-weight people (body mass index ≤25 kg/m^2^) [2]. It is not exclusively the excess of body weight, but more precisely how this excess is distributed, which correlates with cardiovascular risk. In fact, abdominally obese individuals, that is, with an excess of visceral adipose tissue, tend to have higher blood pressure values than individuals with a peripheral body fat distribution [3].

Adipose tissue acts as an endocrine organ by secreting various signaling cytokines, called adipokines, which affect energy metabolism, insulin sensitivity, inflammatory response, and blood flow. Perivascular adipose tissue (PVAT) is the adipose tissue surrounding blood vessels. It was considered until recently only as a passive structural support for the blood vessel, and it was routinely removed for isolated blood vessel studies. Soltis and Cassis [4] demonstrated in 1991 for the first time that PVAT reduced contractions to noradrenaline in rat aorta. This initial description of the anticontractile effect of PVAT was unnoticed until 2002, when Löhn et al. [5] reapproached this issue. These investigators described the inhibitory action of PVAT on aortic contractions to a variety of vasoconstrictors and demonstrated in an elegant bioassay approach that the anticontractile action was induced by a transferable protein factor released by adipocytes [5]. The authors called it adipocyte-derived relaxing factor (ADRF) in analogy to the endothelium-derived relaxing factor (EDRF) described in the 1980s [6].

PVAT is now considered a highly active endocrine organ that releases a variety of adipokines, inflammatory cytokines, and other factors which influence vascular tone in a paracrine way [7–9]. Under physiological conditions, PVAT releases a number of vasoactive substances, such as ADRF [5, 10–13] adiponectin [11], angiotensin-(1–7) [14], H_2_O_2_ [15], leptin [16], and nitric oxide (NO) [17], that elicit a net beneficial anticontractile effect on vascular function and are essential for the maintenance of vascular resistance [7–9].

Since the anticontractile influence of PVAT is directly dependent on its amount [12, 13, 15] and this increases throughout the vasculature in obesity [18–21], it would be conceivable to think that the anticontractile effect of PVAT would be increased in these circumstances. However, it is now known that obesity triggers both structural and functional changes in PVAT which seem to be related to endothelial dysfunction and vascular damage. In fact, in obese patients and animal models of obesity, alterations in the amount and expression pattern of adipokines have been described causing an unbalance in favour of vasoconstrictor and pro-inflammatory substances. It was an approach as simple as leaving the artery intact with its surrounding adipose tissue which has opened a new research line. The aim of this review is to focus on the mechanisms leading to PVAT dysfunction and alteration of its paracrine role in obesity (Table 1).

## 2. The Anticontractile Effect of PVAT Is Lost in Obesity

The loss of the anticontractile effect of PVAT in obesity has been described in several models and conditions (Table 2). Gao et al. [22] demonstrated that the anticontractile effect of PVAT was lost in an animal model of obesity despite higher amounts of perivascular fat. Similarly, New Zealand obese (NZO) mice which have a severe metabolic syndrome and a higher amount of perivascular fat show a reduced anticontractile effect of PVAT [11]. These studies suggest that in obesity, besides to an increased amount of PVAT, there might be changes in the expression pattern of PVAT-derived factors responsible for alterations in vascular function. In this context, a recent study in obese Ossabaw swine [23] described an alteration in the proteomic profile of 186 proteins which correlate with an augmented contractile effect of coronary PVAT and underlying increases in vascular smooth muscle Ca^2+^ handling via *Ca*_V_1.2 channels, H_2_O_2_-sensitive K^+^ channels, and Rho-dependent signaling [23]. The mechanisms involved in increased contractility are reviewed in the following paragraphs.

## 3. Role of ADRF in Obesity

In several studies in obese models [8, 11, 22] and patients [20], the loss of the anticontractile effect of PVAT has been attributed to the downregulation of ADRF. The anticontractile effect of ADRF in physiological conditions has been described in several species [5, 7–13, 24–26] and is mediated by different mechanisms depending on the vascular bed. Both an endothelium-dependent [15] and -independent relaxation [5, 15] have been reported in rat aorta. Endothelium-dependent relaxation is mediated through nitric oxide (NO) release and subsequent calcium-dependent K^+^ channel activation [15], whereas endothelium-independent dilatation is mediated by either the activation of tyrosine kinase pathways and opening of ATP-dependent K^+^ (K_ATP_) channels [5] or by H_2_O_2_ formation [15]. In contrast, ADRF induces an endothelium-independent relaxation through the activation of voltage-dependent K^+^ channels (Kv) in rat mesenteric arteries [12, 13].

An essential question that remains to be answered concerns the nature of ADRF. The identity of ADRF with adiponectin (see below) is controversial. Löhn et al. [5] excluded this possibility, since the anticontractile effect of PVAT is still present in adiponectin knock-out mice (APN −/−) [12]. However, in a recent study, *β*_3_-adrenoceptor stimulation of PVAT under basal, noncontracted conditions releases an adipocyte-derived hyperpolarizing factor which is probably adiponectin [27]. This factor activates AMPK to indirectly open myocyte BK_Ca_ and TRPM4 channels. Additionally, it also liberates NO which also contributes to PVAT-dependent myocyte hyperpolarization [27]. A recent study proposes palmitic acid methyl ester (PAME) as a candidate for ADRF since it also elicits vasorelaxation by opening voltage-dependent K^+^ channels on smooth muscle cells [26]. The identity of ADRF with leptin (see below) has also been discarded, since the lack of functional leptin receptors in the Zucker fa/fa rats did not modify the anticontractile effect of PVAT [5].

## 4. Nitric Oxide and Hydrogen Sulfide: Two Gases in PVAT

**Figure 1:**
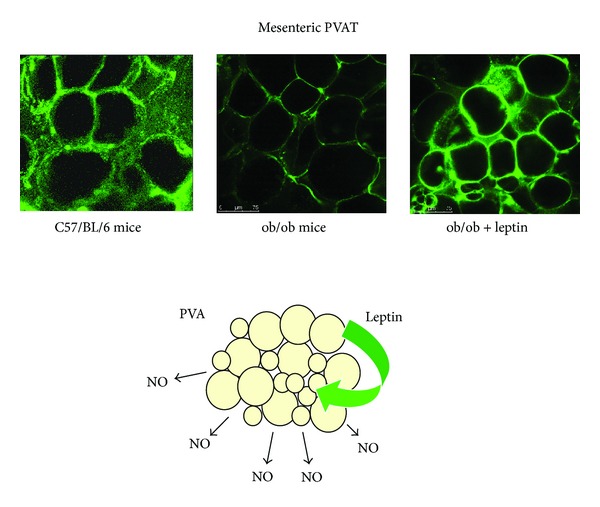
Leptin stimulates nitric oxide (NO) release in perivascular adipose tissue (PVAT). Mice lacking leptin (ob/ob) do not exhibit NO production in perivascular adipocytes. NO release is restored in PVAT after 2-week subcutaneous leptin infusion. Data from [17].

Nitric oxide (NO) release in mesenteric PVAT from C56/Bl6 mice contributes to the enhancement of vasodilator responses [17]. In obese mice, after 32 weeks of HFD, eNOS and NO production in PVAT are downregulated to undetectable levels [28]. Moreover, ob/ob mice lacking leptin do not exhibit NO production in perivascular adipocytes. Interestingly, this is restored in PVAT after 2-week subcutaneous leptin infusion, suggesting that NO release in PVAT seems to be mediated by leptin [17] (Figure 1).

Hydrogen sulfide (H_2_S) production has been demonstrated in rodent aortic and mesenteric PVAT [29, 30] and has been proposed to be ADRF [31]. This gas is synthesized in the cytosol from l-cysteine and is enzymatically oxidized in mitochondria (for a review, see [31]). The effect of H_2_S on blood vessels is dual depending on its concentration, although its net effect seems to be antihypertensive. H_2_S elicits vasoconstriction at low and vasodilation at higher concentrations [32]. Although PVAT-derived H_2_S production has not been studied in obesity, treatment with atorvastatin increases its production preventing mitochondrial oxidation and increasing the anticontractile effect of PVAT [33]. Further research will be necessary to assess the role of H_2_S in obesity.

## 5. PVAT-Derived Adipokine Dysregulation in Obesity

Leptin participates in the regulation of vascular tone (for a review, see [34]). Vascular effects of leptin seem to be the net result of two different actions: (i) a direct vasodilatation depending on an intact and functional endothelium through mechanisms that vary between different vascular beds and (ii) an indirect vasoconstriction through stimulation of sympathetic activity at hypothalamic level [35]. Leptin activates endothelial nitric oxide synthase (eNOS) in aorta [36, 37], whereas it induces the release of endothelium-derived hyperpolarizing factor in mesenteric arteries [37]. *In vivo* experiments have revealed that leptin infusion reduces arterial pressure by increasing NO release [38]. Moreover, leptin exerts an endothelium-independent anticontractile effect on angiotensin II-induced contractions [39].

Leptin released from PVAT has a paracrine role in the regulation of vascular tone. PVAT surrounding rat aorta and mesenteric arteries, as well as the human saphenous vein [40], releases leptin at active concentrations which elicits an anticontractile effect [12, 16]. Moreover, elevated leptin levels in PVAT promote neointima formation independent of obesity and systemic hyperleptinemia [41]. In Ossabaw swine with metabolic syndrome, epicardial PVAT-derived leptin enhancement aggravates endothelial dysfunction via a PKC-*β*-dependent pathway [42]. Similarly, an upregulation of leptin in aortic PVAT is paralleled by a reduced anticontractile effect of PVAT. In a mice model of diet-induced obesity, the increase in leptin levels correlates with a loss in PVAT-derived NO and eNOS [28]. It has to be elucidated, however, if this is a result of a deleterious effect of high leptin levels or of leptin resistance.

Adiponectin induces vasodilatation in rat aorta and in mouse mesenteric arteries through an endothelium independent mechanism involving the activation of Kv channels [11]. Other authors have shown that adiponectin increases NO release from vascular endothelial cells in culture [43, 44]. Moreover, adiponectin seems to preserve endothelial function through inhibition of endothelial cell activation [45] and synthesis of inflammatory markers [46]. The first evidence for the paracrine vasodilator effect of PVAT-derived adiponectin was demonstrated by Greenstein et al. [20]. Incubation with an adiponectin type I-receptor blocking peptide entirely abolished the anticontractile properties of PVAT in human subcutaneous and in rat arteries, suggesting that adiponectin is a physiological modulator of local vascular tone by increasing NO bioavailability.

Since eNOS is the final step for leptin- and adiponectin-induced NO release, alterations in this enzyme might be related with obesity-related endothelial dysfunction. In this context, an impairment of eNOS-mediated vasodilatation through a downregulation of the AMPK/mTOR pathway in obese rats has been shown [19]. Thus, vascular dysfunction in obesity is both the result of adipokine disbalance as well as of a downregulation of their signalling pathways.

## 6. Oxidative Stress and Inflammation in PVAT Are Increased in Obesity

One proposed mechanism to explain the loss in the anticontractile effect of PVAT is the increase in oxidative stress observed in obesity. Reactive oxygen species (ROS), such as superoxide anion or hydrogen peroxide (H_2_O_2_), play an important role in PVAT-mediated modulation of vessel function. NADPH oxidase, which represents the major source of superoxide anion in the vasculature, is also expressed in PVAT of rat mesenteric arteries [15]. In these vessels, PVAT-derived superoxide anion enhances the arterial contractile response to perivascular nerve stimulation involving activation of tyrosine kinase and MAPK/ERK pathway. Superoxide anions are rapidly converted by superoxide dismutases (SODs) to H_2_O_2_, which is a cell-permeant and highly stable ROS [47]. Expression of the three SOD isoforms, the copper-zinc SOD (Cu/Zn-SOD), the manganese SOD (Mn-SOD), and the extracellular form of Cu/Zn-SOD (ec-SOD), has been detected in mice mesenteric PVAT [28].

H_2_O_2_ has been shown to be a vasoactive substance that induces both contractile and relaxant responses on blood vessels by different mechanisms depending on the vessels type, the contractile status, its concentration, and the animal species [47]. Contractile effect mediated by H_2_O_2_ is due to direct cyclooxygenase activation and to an increase of intracellular Ca^2+^ [48, 49]. Interestingly, it has recently been suggested that extracellular H_2_O_2_ can enter vascular smooth muscle cells stimulating Nox1 oxidase and superoxide anion production [50]. H_2_O_2_ also induces endothelium-dependent relaxation as a result of an increased NO release secondary to endothelial K^+^ channel activation [48]. Moreover, H_2_O_2_ induces endothelium-independent relaxation through (i) direct opening of smooth muscle K^+^ channels by oxidation of their cysteine residues, as well as (ii) by direct activation of smooth muscle soluble guanylate cyclase (sGC) [15, 51].

Since superoxide anion promotes vessel contraction, while H_2_O_2_ induces its relaxation, the final outcome will depend on their relative PVAT production/release balance, and activity of SODs in PVAT might be crucial [28]. Ketonen et al. [52] showed in C57/Bl6 mice fed a very high fat diet (60% cal from fat) for 8 weeks that endothelial-dependent relaxation was due to an increase in PVAT-derived oxidative stress characterized by the enhanced production of superoxide anion and hydrogen peroxide. Another study in mice fed a long-term high fat diet (45% cal from fat 32 weeks) showed that mesenteric bed endothelial dysfunction was aggravated in the presence of PVAT [28]. An increase in NADPH oxidase activity and in superoxide anion production, together with a decrease in ecSOD expression and total SOD activity, was found in PVAT. These changes were accompanied by a decrease in eNOS expression and NO production in PVAT from these obese mice [28]. Similarly, in NZO mice, an impaired H_2_O_2_ production in PVAT, as a consequence of increased *·*O_2_^−^ formation and decreased SOD expression, contributes to vascular dysfunction through reduced anticontractile effects [53]. Ossabaw obese swine, H_2_O_2_-mediated vasodilatation was markedly attenuated by the presence of coronary PVAT [24].

An interesting issue which deserves future investigation is the impact of the fatty acid (FA) composition in the diet to the increase in oxidative stress in PVAT. A fructose-rich diet decreases polyunsaturated FA, increasing saturated and monounsaturated FA in PVAT [54]. These changes in FA composition are paralleled by a decrease in antioxidant enzymes, a reduction in glutathione content, and alterations in vascular function.

Obesity is also associated with a state of chronic low-grade inflammation that can be detected both systemically and within specific tissues. An increase in inflammatory cytokines has been shown in PVAT surrounding small arteries from obese subjects with metabolic syndrome correlating with an elevated oxidative stress [20]. High-fat diet-induced obesity promotes a marked proinflammatory shift in the profile of secreted cytokines and chemokines which is associated with oxidative stress in PVAT [55]. PVAT of NZO mice also exhibits inflammation and an increase in oxidative stress, leading to endothelial dysfunction, the latter as a result of decreased NO and enhanced superoxide generated by uncoupled endothelial NO synthase [53].

Moreover, HFD causes an increase in expression of leptin and MIP1alfa correlating with a decrease in adiponectin, PPARy, and FABP4 [56]. This is paralleled by an enhanced CD3 expression in PVAT with no changes in CD68 levels [56]. In another study, diet-induced obesity increased mesenteric PVAT macrophage content and vascular oxidative stress in mice [57]. Human adipocytes show an increase in proinflammatory state (IL-6, IL-8, and MCP-1) and reduced adipocytic differentiation [56]. These data suggest that inflammatory cytokine release by PVAT could attract macrophages to the depot further aggravating inflammation and PVAT dysfunction. The key role of inflammatory cells in PVAT-aggravated endothelial dysfunction was demonstrated in mice lacking P-selectin glycoprotein ligand-1 (Psgl-1), an inflammatory adhesion molecule enabling the recruitment of leukocytes to the endothelium. Psgl-1 deficiency prevented PVAT inflammation and endothelial dysfunction [57]. In contrast to the abovementioned findings, Fitzgibbons et al. [58] have proposed that mice thoracic PVAT shows a very low inflammation after 13 wk of HFD, probably due to its similarity with the brown adipose tissue phenotype. This study suggests the attractive possibility of promoting a BAT phenotype in PVAT which might have preventive effects in vascular disease development.

## 7. Role of Hypoxia in Obese PVAT

It is well known that the increase of the adipocyte area and mass that occurs in obesity leads to hypoxia [59–61] (Table 3). However, there are controversial results about the role of hypoxia in PVAT. Maenhaut et al. [61] demonstrated that hypoxia enhances relaxation of mouse aorta in presence of PVAT independently of the vasoconstrictor agent. This effect is mediated by a basally released factor which meets the criteria for ADRF [7, 10]; that is, (i) it acts through K_ATP_ channels, (ii) it is independent of NO and a functional endothelium, and (iii) its similarity with lactate, CO, H_2_S, prostanoids, adenosine, leptin, or adiponectin has been excluded. In obese patients, however, hypoxia abolishes the anticontractile effect of PVAT and has been linked to oxidative stress and increased inflammation in PVAT [20].

## 8. Role of Aging in Obese PVAT

Obesity in elderly individuals is increasing at alarming rates, and there is evidence suggesting that this population is more vulnerable to the deleterious cardiovascular effects of obesity than younger individuals [62]. The paracrine effect of young and aged adipocytes on vascular smooth muscle cell proliferation has been described by Barandier et al. [63]. These authors nicely showed that proliferative effects of adipocyte-conditioned medium were significantly increased in 24-month-old and in HFD WKY rats versus young 3-month-old animals. Aging also exacerbates endothelial dysfunction and vascular inflammation in HFD mice due to an increase in oxidative stress, inflammation, and macrophage infiltration in periaortic adipose tissue [55]. These studies suggest that that there is a synergy between age and obesity-related alterations in PVAT damaging the vascular wall. However, more studies will be necessary to deeper characterize the mechanisms and impact of aging on the paracrine effects of PVAT in obesity.

## 9. Impact of Weight Loss on PVAT Dysfunction

Weight loss by different approaches significantly correlates with improvements in blood pressure levels, left ventricular mass, exercise capacity, and glucose tolerance [64]. Bariatric surgery by gastric bypass has been shown to reduce white adipose tissue inflammation by inducing significant reductions in macrophage content, MCP-1, and hypoxia inducible factor-1*α* [65]. Interestingly, bariatric surgery also reverses the obesity-induced damage to PVAT anticontractile function by reducing adipocyte hypertrophy, PVAT inflammation and increasing both PVAT-derived NO and adiponectin availability [66]. This study opens a new approach for the management of vascular damage associated to PVAT dysfunction.

## 10. Overweight versus Obesity: Adaptative Changes in PVAT 

After onset of high fat, feeding alterations in PVAT develop in a sequential manner. Early stages of diet-induced obesity (DIO) are characterized by increased adiposity (overweight rather than obesity) and moderate hyperleptinemia but preserving peripheral responsiveness to leptin, as well as normal postprandial values of adiponectin, insulin, glucose, triglycerides, and free-fatty acids [67]. It is well known that fat-enriched diets trigger initial compensatory mechanisms in adipose tissue aimed at preventing organ damage and lipotoxicity [68]. In this context, our group has recently shown that a moderate enlargement of PVAT during early DIO correlates with NO overproduction in this tissue and an improvement of vascular function [28].

**Figure 2:**
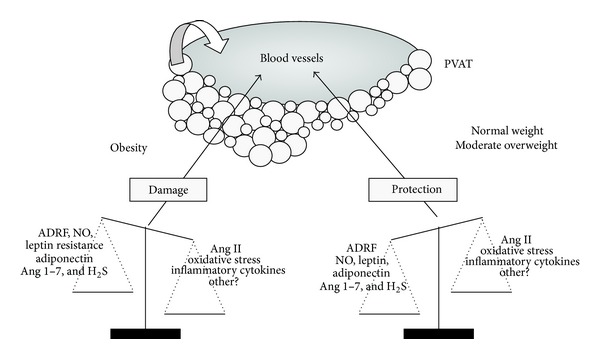
Hypothesis of the role of PVAT and PVAT-derived adipokines in health and obesity.

We thus suggest that the consequence of PVAT enlargement in response to a HFD might be dual (Figure 2). Initially, a moderate PVAT enlargement might be beneficial, being an adaptative response probably aimed at protecting vascular function in situations such as moderate overweight, pregnancy, or hibernation. In established obesity, however, changes in PVAT amount and in the expression pattern of adipokines and other PVAT-derived factors might shift the paracrine influence of PVAT from a net anticontractile effect to a prooxidant, proinflammatory, and contractile environment. This unbalance towards a predominance of vasoconstrictor and inflammatory factors in obesity could provide the link between obesity, cardiovascular functional, and structural alterations and cardiovascular diseases.

Several questions regarding PVAT dysfunction need to be addressed in the next years: (1) to identify new PVAT-derived factors that reach the vascular wall, (2) to determine the time point when the balance shifts from a protective to a deleterious paracrine effect of PVAT during weight gain, (3) to characterize the interplay between different adipokines (actions and time-course), and (4) to assess the effects of dietary or pharmacological interventions on PVAT-derived adipokine expression profile and vascular function. A better understanding of PVAT dysfunction may lead to new approaches in the management of cardiovascular risk prevention in obesity.

## Figures and Tables

**Table 1:** Mechanisms of PVAT dysfunction.

(i) Increase in adipocyte size and PVAT amount	
(ii) Hypoxia	
(iii) Aging	
(iv) Leptin/adiponectin dysregulation	
(v) Loss of anticontractile properties	
(vi) Loss of eNOS and NO	
(vii) Increase in oxidative stress	
(viii) Increase in inflammatory response	

**Table 2:** Loss of anticontractile effect of PVAT in obesity.

Study reference	Vessel	Type of PVAT	Species	Obesity
[20]	Small arteries (100–150 *µ*m)	Subcutaneous gluteal	Human	Obese patients
[28]	Mesenteric arteries	Mesenteric	C57BL6 mice	DIO (32 w)
[11]	Mesenteric arteries	Mesenteric	NZO mice	
[53]	Mesenteric arteries		NZO mice	
[19]	Mesenteric arteries	Mesenteric	Rat	DIO (6 mo)
[19]	Aorta	Periaortic	Rat	DIO (6 mo)
[22]	Aorta	Periaortic	Rat	Perinatal nicotine adm
[42]	Coronary	Epicardial	Ossabaw obese swine	DIO (20 w)
[23]	Coronary	Epicardial	Ossabaw obese swine	DIO (6–12 mo)

**Table 3:** Increase in PVAT amount and adipocyte size in different models of obesity.

Species	Model	PVAT	Adipocyte size	Study reference
Wistar rat	HFD (6 mo)	Aortic	Increase	[19]
Wistar Kyoto rat	HFD (3 mo—60% cal from fat)	Aortic	Increase	[63]
Zucker fa/fa rat		Aortic	Increase	[63]
C57Bl6 mice	HFD (2 w—42% cal from fat)	Aortic	Increase	[56]
C57Bl6 mice	HFD (13–20 w—45% cal from fat)	Thoracic aortic	No change	[58]
C57Bl6 mice	HFD (8 w—45% cal from fat)	Mesenteric	Increase	[17]
C57Bl6 mice	HFD (32 w—45% cal from fat)	Mesenteric	Increase	[28]

HFD: high fat diet; mo: month; w: weeks.
